# A Conceptual Framework for Action Research on the Sociocultural Aspects of Pregnancy and Postpartum Care among Brazilian Indigenous Women

**DOI:** 10.1177/08445621261474578

**Published:** 2026-08-04

**Authors:** Margareth Santos Zanchetta, Leandro Da Silva De Medeiros, Ingrid Lima Silva, Dirce Stein Backes, Barbara Chyzzy, Fábio Prado Saldanha, Juliana Silveira Colomé

**Affiliations:** 1 Daphne Cockwell School of Nursing, 7984 Toronto Metropolitan University, Toronto, Canada; 2 Graduate Program in Maternal-Child Health, Franciscan University, Santa Maria, Brazil; 3 School of Nursing, 67892 Federal University of Amazonas, Manaus, Brazil; 4 Économie et innovation sociale, 647717 Université d'Ontario francais, Toronto, Canada

**Keywords:** action research, Brazilian Indigenous women, conceptual framework, health promotion, pregnancy-puerperium context

## Abstract

**Background:**

Brazil has recognized the need to investigate the health context of Indigenous Peoples by (a) collecting data on service coverage, barriers, needs, and gaps in access to health care; (b) developing, funding, and implementing plans and strategies for multilingual service delivery (c) reducing gender, social, and cultural inequalities as well as geographical barriers that hinder equitable access to quality health services; and, (d) incorporating an intercultural-intersectoral approach in the development of policies and inclusive-participatory research.

**Purpose:**

Propose an original conceptual framework for action research on the socio-cultural aspects of pregnancy, childbirth, and the postpartum cycle among Brazilian Indigenous women.

**Results:**

An online consultation with healthcare professionals was conducted (April-June 2024) using audio recorded narratives to gather information about the health of pregnant and postpartum Indigenous women. The Canadian model of population health promotion guided information analysis. Fourteen healthcare professionals with experience working in four Brazilian states and with 11 ethnic groups were consulted. A total of 23 modifiable situations were identified, and an original conceptual framework was developed that encompasses the following health promotion areas as modifiable situations: structure of the healthcare network; administrative barriers in healthcare management; operationalization of care delivery; and clinical and social contexts.

**Conclusion:**

This article conceptualizes areas for innovative and socially responsive solutions to amend vulnerabilities in Brazilian Indigenous maternal health. The conceptual framework proposed offers a useful operational foundation for designing future interventions in Indigenous maternal health and for strengthening action research as a transformative methodological strategy in contexts marked by persistent historical and social inequalities.

## Background

Self-determination is a core principle of international human rights law, upheld by numerous United Nations agreements including the International Covenant on Economic, Social and Cultural Rights (United Nations [UN], 1966a) the International Covenant on Civil and Political Rights (UN, 1966b), and the Vienna Declaration and Program of Action (UN, 1993). These international agreements guarantee Indigenous Peoples’ right to maintain their health, religious, and medicinal practices, including access to health services, without any discrimination. Maternity, labor and childbirth have a unique meaning to Indigenous Peoples, whose acknowledgement is part of their human rights (UN, 2008). In addition, the World Health Organization [WHO] (2023) approved a resolution on the health of Indigenous Peoples as a worldwide priority issue with the goal of advancing health systems to promote specific actions for this population. This WHO resolution establishes Indigenous Peoples’ political status and affirms their right to freely pursue their economic, social, and cultural development, and safeguards collective rights that are indispensable for their existence and well-being (UN, 2008).

Indigenous health has global relevance since 476 million Indigenous Peoples live in 90 different countries (about 6% of the world's population and 19% of people living in extreme poverty), whose life expectancy is up to 20 years lower than that of non-Indigenous Peoples worldwide (Pires et al., 2024). Brazilian statistics (Instituto Brasileiro de Geografia e Estatística [IBGE], 2024) indicates that the Indigenous population of Brazil is 1,693,535, corresponding to 0.83% of its total population, with illiteracy rates in urban areas at 15.05% and 20.8% in Indigenous lands. The population of 860,000 Indigenous women is distributed across Indigenous lands (48.7%) and urban areas (51.3%) (IBGE, 2024).

It is important to highlight obstacles to the realization of Indigenous Peoples’ human rights with respect to health care, and specifically maternal and postpartum care due to structural discrimination and lack of representation and inclusion in decision-making processes. Consequently, these issues impact the quality of services received by Indigenous women and girls and their general living conditions (Organização Pan-Americana da Saúde, 2019). Brazil is a signatory to these agreements and has implemented numerous initiatives and projects for medical-social assistance, development, and academic research to improve the quality of life for Indigenous Peoples. The most ethically and culturally respectful and appropriate approach is one that gives Indigenous Peoples the leading role in identifying issues that should be prioritized and addressed.

Brazil has recognized the need to investigate the health context of Indigenous Peoples through various actions: (a) collecting data on coverage and obstacles, needs, and gaps in access to health services; (b) developing, funding, and implementing plans and strategies focused on the multilingual delivery of services enhancing cultural responsiveness; reducing gender disparities and improving recognition of gender specificities; eliminating or reducing cultural, social, and geographical barriers; and improved responses to public health emergency situation; (c) reducing gender, social, and cultural inequality and geographical barriers to equitable access to quality health services provided in Indigenous lands; (d) incorporating an intercultural and intersectoral approach in the development of policies on the health of Indigenous Peoples; and (e) adopting an inclusive and participatory approach in the development and implementation of research and development (WHO, 2026).

### State of Knowledge

#### Social Determinants of Health: Influences on Indigenous Maternal Health

Research has pointed to several synergistic social determinants of health (SDH) and their roles in the Indigenous maternal health in Brazil as presented in the upcoming paragraphs. Current state of knowledge based on Brazilian scholars’ work has sparked scientific curiosity for the development of some directions for health professionals’ future engagement with Indigenous communities (as strategic social actors) in promoting practices and interventions to improve maternal health while at the same time respecting local cultural traditions (Silva et al., 2024).

Access to primary health care (PHC) services and hospital units by pregnant Indigenous women, especially those living in remote areas, is related to several interconnected sociocultural factors: (a) separation from the nuclear family, resulting in difficulties in caring for younger children; (b) reluctance to seek services due to knowledge of negative experiences reported by other women; and (c) suspicion of Western practices that disregard beliefs and traditions shaping the Indigenous conception of health and disease (Silva et al., 2024).

Culture plays a fundamental role in Indigenous maternal health, influencing care practices, access to health services, and adherence to treatments, which can impact the prevention and management of obstetric complications, such as hypertensive disorders, hemorrhages, and infections (Garrafa et al., 2024). Indigenous Peoples also have distinct cultural perspectives on pregnancy, childbirth, and the postpartum period, and these are expressed in various health practices and habits. For example, among the Kaiabi people living in the Middle Xingu, the diet during pregnancy is based on fish and beiju (a pancake made of cassava flour), contributing to the high prevalence of anemia. The maternal-infant nutritional status is directly influenced by beliefs related to food. There is a prohibition against looking at certain animals or consuming specific types of fish, restricting the diet of pregnant women, new mothers, and even fathers (Nafakua et al., 2024). Among the Guarani people, both parents, after conception, follow a specific diet avoiding foods that they consider harmful to the newborn's health (Ministério da Saúde [MS], 2016a).

For the Kaingang people, the practices passed down by the elders ensure care and protection for the child among various community members (MS, 2016b). Prenatal care provided by Kaingang midwives involves a specific diet, baths, and teas that prepare the pregnant woman for the child's birth. In addition, there is a series of exercises and activities aimed at strengthening the abdominal muscles, assisting in preparation for childbirth. During a woman's first childbirth, she usually goes to the fields accompanied by her mother or another experienced woman. In subsequent births, it is common for her to go alone and return with the newborn. It is believed that the fetus should continue to be nourished by male semen, making sexual relations during pregnancy essential for the strengthening of the fetus (MS, 2016b; Pereira, 2016).

Home birth is still widely practiced and carries cultural importance and symbolism. Boer et al. (2024) identified that Indigenous women prefer to give birth in the village, due to the presence of other women (mother, mother-in-law, family midwife) as well as other close individuals. The birth is also marked by cultural rituals, such as among the Munduruku people, where the cloths used during childbirth, especially those with bloodstains, are considered dangerous because they are believed to attract enchanted beings from the underwater world, such as river dolphins. To avoid these risks, these clothes are buried or burned, reinforcing the relationship between spirituality and protection (Dias-Scopel, 2018). On the other hand, the growing influence of hospital medicine, whether due to the guidance of the healthcare team or awareness of obstetric complications that require specialized assistance, because Indigenous Peoples recognize hospital role in improving health outcomes due to safety matters (Dias-Scopel, 2018).

In the postpartum period, among the Guarani people, women remain in home rest for three to five days, avoiding sun, wind, and physical exertion (Claudino et al., 2024; Velasquez et al., 2024). Among the Munduruku people, women enter a period of liminality—an intermediary condition between the transition from being a woman to a mother—in which the woman's body, blood, and health are preserved by prohibiting certain activities and encouraging seclusion, encompassing the mother, father, and newborn. There is a strict set of prescriptions known as “postpartum confinement,” “confinement of the birthing woman,” or simply “confinement,” which also includes dietary restrictions (Dias-Scopel, 2018).

#### Public Policies: Issues and Challenges

Despite the development of social and health policies aimed at Brazil's Indigenous population in the last two decades, maternal health indicators remain concerning. The problem is further compounded by inconsistencies and delays in the official statistical data on the clinical context in PHC and the health of Indigenous women (Alencar & Araújo, 2024). For instance, research has shown that difficulties in obtaining reliable information regarding the spectrum of underlying issues related to maternal mortality among Indigenous Peoples is common. These women's survival for the preservation of ancestral knowledge and cultural continuity should be a national priority for the promotion of Indigenous health and respect for women's human rights as a social indicator of sustainable development in Brazil (UN, 2015). Research between 2015 and 2021 revealed that postpartum hemorrhage was the leading cause of maternal deaths among Indigenous women compared to eclampsia among non-Indigenous women (Garrafa et al., 2024). Maternal mortality among Indigenous women was 115 deaths per 100,000 live births, while among non-Indigenous it was 67 per 100,000 live births (Garrafa et al., 2024).

The prenatal healthcare of Indigenous women is coordinated by the Indigenous Health Care Subsystem, interconnected with the Unified Health System (SUS) network and operationalized through 34 Special Indigenous Health Districts, under the management of the Special Secretariat for Indigenous Health (Silva et al., 2024). These authors found that in more remote Indigenous communities and villages, geographical and structural barriers hinder the provision and continuity of prenatal care. Difficulty in directly accessing health services impacts women's ability to engage in prenatal consultations within the first trimester of pregnancy to detect any abnormalities for mother and fetus (Silva et al., 2024).

### Responsive Research Planning

Better understanding of the geographical and structural barriers can be achieved through qualitative research approaches. These methods have the potential to reveal the multidimensional particularities of Indigenous maternal mortality, especially with respect to social disparities and inequalities in health care. It is important to uncover the local knowledge to better understand the social processes surrounding motherhood. Research should strive to facilitate collective construction and knowledge development by and for the communities themselves, while avoiding researcher-centered top-down approaches.

Action research (AR) is a particularly powerful qualitative approach that allows for the collective construction of solutions that respect traditional knowledge and promote the autonomy of communities (Reason & Bradbury, 2008), which can be fit in the pursuit of better Indigenous maternal health conditions. Action research in socially vulnerable communities can provoke sustainable changes and produce meaningful knowledge, the active involvement of the community, and the feasibility of the intended changes. Reflexive engagement through AR with community members to identify various forms of assets can bring forth the strengths and inherent needs of the community, especially in communities that have suffered various forms of historical injustice as lived in Brazil (Zanchetta et al., 2014). Since AR allows and values the active participation of communities in decision-making for the implementation of research, it also questions reality and believes in the potential of social actors to transform local reality (Bergamaschi & Pinheiro, 2021; Busarello & Sawaia, 2022) in an interactive and reflective process to inform future actions and improve health practice (Moura Junior & Silva, 2024). Therefore, it is necessary to provide visibility to the experiences of Indigenous women (Pantoja, 2021) through AR, whose potential contribution to scientific advancement in the Indigenous context is considerable in its dynamic-methodological model.

As integral to the process of planning AR, researchers should work collaboratively with community partners to identify issues that would warrant joint action. This participatory process not only ensures that research is grounded in community priorities but also fosters mutual accountability and shared ownership of outcomes. Engaging in such collective action would lead to a better understanding of community assets and potential for change, which is central to a more human-centered form of development (Hancock, 2001).

## Purpose

This article reports the information gathered in a community anonymous consultation with health professionals experienced in Indigenous maternal health issues on the state of Indigenous maternal healthcare in Brazil. The consultation aimed to inform exclusively the design of the conceptual framework to guide a future implementation of AR related to the socio-cultural aspects of pregnancy, childbirth, and the postpartum cycle among Brazilian Indigenous women. By its purely conceptual intention, this work had two specific aims: (a) to provide a multidimensional picture of the challenges, barriers, and difficulties, to be encountered in the AR implementation; and (b) to inspire possible participatory actions with Indigenous communities and other social actors for the desired social changes.

## Baseline Conceptual Framework

A range of SDH, such as cultural values, care practices, biological sex, health literacy, genetic makeup support networks, child development, personal health practices, and psychological coping mechanisms play an essential role in the health of Indigenous Peoples. These factors influence not only individual well-being but also collective well-being, which encompasses spiritual, mental, physical, and emotional dimensions throughout the entire life cycle. To understand this systemic perspective is crucial for the formulation of culturally appropriate and effective health promotion strategies (Hamilton & Bhatti, 1996). Thus, actions and interventions aim at improving the well-being of Indigenous people must consider the interconnectedness between traditional knowledge and health policies, ensuring approaches that respect and strengthen the autonomy and well-being of these populations.

The design of the consultation was based on the Canadian Population Health Promotion Model (PHPM) (Hamilton & Bhatti, 1996). This model includes the dimensions of health promotion and SDH. The PHPM proposes integrative, comprehensive, and simultaneous actions for health promotion to optimize the SDH, namely: strengthening community action, creating supportive environments, developing personal competencies, redesigning public health systems, and proposing healthy public policies, each of which make health promotion feasible. The PHPM also indicates interventions to promote health at the individual, family, community, sector/system, and societal levels, linking the individual with their ancestry, gender, life, and experiences of illness. The relevance of this model in proposing a conceptual framework is evident in the philosophical similarities and harmony between the Canadian PHPM and that of the Brazilian SUS, mainly at the PHC level. In other words, Brazil's SUS shares philosophical grounds with the Chart of Ottawa which supported the conception of the Canadian PHPM. Such philosophical harmony has allowed for Canada-Brazil joint initiatives since 1980s in PHC.

Moreover, culture as a SDH can be understood as applicable to many ethnic groups including the Brazilian Indigenous and those of Canada. Furthermore, WHO (2026) in their declarations, definitions of health and other actions have been intensively active in the transnational application of health promotion models through knowledge transfer, focusing on theoretical, conceptual, empirical and experiential contents.

## Method

Design: This consultation was informed by the rapid participatory appraisal of community health needs approach (Annett et al., 1995) applying its two modalities: (a) participatory rural appraisal—in which planners and the community are partners in the collection and analysis of information and in proposed actions; and (b) rapid ethnographic appraisal—using anthropological methods to assess community perceptions, beliefs, and practices regarding health, diseases, and health interventions.

The community consultation was not a research activity per se. However, it is necessary to inform that in Brazil (National Health Council, Resolution n. ° 510/ 2016 (MS, 2016a), any type of humanities and social sciences inquiry whose data collection does not identify sociodemographic information of the respondent does not require review and approval by an ethics review committee.

### Identification of Prospective Informants

Prospective informants were identified among those HCPs and researchers with known experiences in the Indigenous health. They belonged to the authors’ professional networks and of other HCPs who had information about their own colleagues in their social media networks. An invitation through WhatsApp messaging was sent to them. No recruitment and sampling processes were applied in this community consultation with professional experts.

### Collection of Information

The online consultation took place via WhatsApp from April to June 2024. Informants responded orally to the questions by recording themselves and sending their electronic responses as a short audio narrative. They responded to the following questions: (1) What do you know about the health situation of pregnant and postpartum Indigenous women in your catchment area? (2) What do health professionals who assist the villages think about the health situation of pregnant and postpartum Indigenous women in your catchment area? and (3) What could be done to improve the health situation of pregnant and postpartum Indigenous women? Informants responded freely to the guiding questions, providing details according to their own judgment, allowing the planning team to grasp the diversity of lived experiences in the many different locations of their professional practice.

### Information Analysis

The audio recordings were transcribed and anonymized by the second and third authors and the first author initially identified 47 multidimensional situations that could be considered components of the sociocultural specificities in the pregnancy-puerperium cycle of Brazilian Indigenous women. These situations could also refine the future focus of the research questions and areas of investigation during the fieldwork.

The transcribed narratives were subjected to an adapted content analysis method to identify the meaning proximity of descriptive words, create categories of meaning, analyze polarized themes, and identify associations between conceptual categories (Bardin, 2007). This work allowed the retrieval and organization of words in semantic categories. An in-depth work was not possible due to the scarcity and superficiality of the spontaneous and unguided narratives**.**

Detailed information on the analytical procedures follows: (a) extensive reading of the narratives by three first co-authors; (b) identification of contents regarding personal view of Indigenous health's context, administrative infrastructure, administrative and cultural barriers, practice challenges, persistent difficulties and changes to be implemented; (c) separation and classification of contents as per types of SDH and the areas of HP as per the Canadian PHPM (Hamilton & Bhatti, 1996); (d) identification and selection of contents, naming them as either modifiable situations or immutable factors; (e) classification of selected contents as description of a SDH's area (f) use each SDH as a conceptual categories; rearrangement of these contents to be addressed according to areas of integrative health promotion as proposed in the Canadian PHPM; (g) allocation of sets of SDH as per areas considered as conceptual themes; and (h) design the model representing the original dynamics of the sociocultural specificities in the pregnancy-puerperium cycle of Brazilian Indigenous women in their original context of life. The confirmation of accuracy of the information collected with informants was not conducted due to their unavailability 8 months after the consultation.

#### Information Collected Through Consultation

A total of 14 professionals participated in the consultation, 7 of which defined their role in the field of health services. These included nurses (obstetrician, municipal health secretary), a biologist, a medical entomologist, a nutritionist, and a deputy health secretary. The short narratives, which averaged one minute and 58 s, were sent in 37 audio files because the informants sent more than one audio file. The narratives focused on the professionals’ impressions and experiences in the context of Indigenous health in the states of Amazonas, Amapá, Mato Grosso, and Rio Grande do Sul, and involved 11 ethnic groups: Apurinã, Guarani, Kaingang, Kaixana, Kambeba, Kokama, Madija, Kulina, Parintintin, Pirahã, and Ticuna.

The gathered information was read in-depth and arranged in two sets: immutable factors and modifiable situations. Immutable are those factors (n = 24) that any PHC interventions could modify such as: (a) language barriers faced by Indigenous women when interacting with HCPs who solely master the Portuguese language; (b) multiple Indigenous languages to be mastered by non-Indigenous and sometimes even by the Indigenous HCPs; (c), women's reproductive rights as secure by Brazilian legislation, including pregnancy in preadolescents and adolescents and late pregnancies; (d) cases of Indigenous multiple pregnancies with short intervals between them; (e) culturally assigned social roles based on power and male biological sex (the unquestionable power of the Indigenous leader and the husband); (f) resistance by husbands to seeking care, overcome fear of sterilization, and hospital delivery), and (g) government infrastructure (lack of transportation due to poor road conditions, lack of fuel, lack of internet access). Altogether these 24 factors were excluded from the perspective of implementing PHC interventions due to their lack of technical feasibility and most of all for their potential to threaten Indigenous cultural self-determination and autonomy.

Furthermore, 23 modifiable situations were identified (see Display 1) and were grouped into four thematic areas: (a) structure of the healthcare network; (b) administrative barriers in healthcare management; (c) operationalization of care delivery; and (d) clinical and social context (see Figure 1). Modification of situations could occur through the actions of various social actors such as Indigenous leadership, Indigenous women, husbands, local managers of primary care services, health professionals, Indigenous health agents, and other social actors (see Display 1).

**Figure 1.**
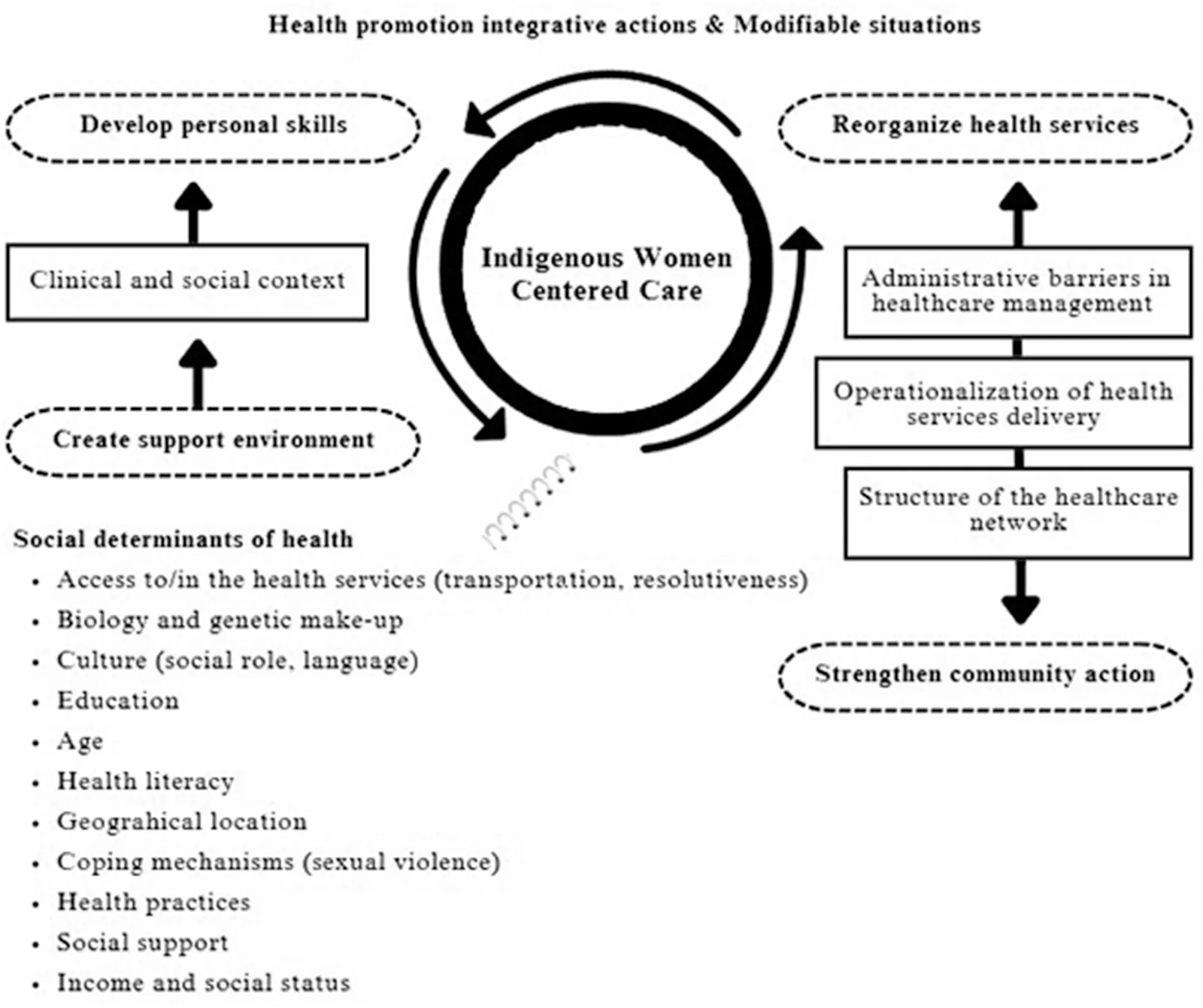
Original Conceptual Framework - Health Promotion Integrated Actions and Modifiable Situations.

**Display 1.** Modifiable Situations and Possible Social Actors for Collaborative Action.

Structure of the healthcare network	Administrative barriers in healthcare management	Operationalization of care delivery	Clinical and social contexts
Devaluation of Indigenous culture due to prejudice from non-Indigenous health professionals^d,e,f,g^	Scarcity of medications^d,g^	Difficult enrollment of Indigenous pregnant women^a,c,d,f^	Resistance of women to hospital childbirth^a,b,c^
Insufficient education in maternal and child health care among Indigenous and non-Indigenous health professionals^d,e,f^	Restricted access to essential exams, diagnostics, and prenatal care, and delay in results delivery^d,e,f^	Compromised systematization of prenatal care in reserves and villages^a,d,f^	Women's arrival at healthcare units in the active phase of labor^a,b,c^
Interruption of monitoring, active search, and continuity of care for Indigenous pregnant and postpartum women ^a,d,e,f^	Required hospitalization for the access to diagnostic exams at the Indigenous Health Support House^d^	Interruption and refusal of prenatal care^a,b,c,d,f,g^	High rates of urinary infection, puerperal infection, and umbilical stump infection^a,b,c,e,f,g^
Paucity of specific medications and health guidance in primary healthcare for Indigenous women^a,d,e,f,g^	Disarticulation in the screening and scheduling process^d,e,f^	Culture of silence on issues of reproductive health and sexual and gender-based violence^a,b,c,f,g^	Men have predominance of power regarding reproductive health decisions^a,c^
Lack of transportation in obstetric emergencies and lack of access to intensive care ^a,d,e^	—	Insufficient postpartum, neonatal, and child care^a,c,d,e,f^	Limitations within women's social support network inhibiting them from seeking and accessing services ^a,b,c,g^
Insufficient dental care^d,e,g^	—	Rupture of bonds among professionals and pregnant women^a,b,c,d,e,f^	Food insecurity of the breastfeeding mother^d,e,f,g^
—	—	Absence of standards for referrals and counter-referrals of services in secondary and tertiary care^d^	—

Note: Collaborative action among social actors to address modifiable situations Legend: ^a^Indigenous leadership; ^b^Indigenous woman; ^c^Husband; ^d^Manager; ^e^Health professional; ^f^Indigenous health agent; ^g^Other social actors (community, family members, friends, religious leaders, volunteers, etc.).

The actions of such actors in future collaborative actions would get the future phase of problem-solving in our action research, whose goal refers to mitigating, resolving, and modifying the identified situations (see Display 1). The subsequent action research phase will also identify other health-related problems.

It is important to highlight that the development of new public policies was not mentioned by informants during the consultation. This can be justified by the informants’ professional practice in the scope of Brazil's National Policy for Care of Indigenous Peoples, implemented in 2002 (MS, 2002) (Silva et al., 2024). Thus, the conceptual model does not include proposals for the creation of public policies targeting Indigenous women's health. Furthermore, it is considered that the absence of the mentioned content, may reflect the existing national capacity in developing guidelines and health policies.

## Original Conceptual Framework

Following analysis of the modifiable situations presented in the Display 1, the authors developed an original conceptual framework which draws on the Canadian PHPM (Hamilton & Bhatti, 1996). It is necessary to inform that the third and fourth co-authors acted as natural experts in Indigenous health contexts as per their professional experience and cultural immersion in Indigenous communities belonging to three of five of Brazil's geographical regions. Such expertise guided the team in the analytical work during the identification of immutable factors and modifiable situations.

Figure 1 was designed using the popular, well-known image of the Canadian PHPM. Each element identified in the analysis was distributed to the area displayed in the image. The figure depicts the identified sociocultural specificities in the pregnancy-puerperium cycle of Indigenous women, contextualized in four of the five areas of integrative actions (e.g., strengthening community action, creating supportive environments, developing personal competencies, redesigning public health systems, and proposing health public policies) proposed in the Canadian PHPM and influenced by the relevant SDH in the life context of Indigenous women. The proposed framework is grounded in philosophical ideas of community-centered care, which is consonant with AR, and whose protagonism relies on the participants instead of the researcher. Therefore, the joint actions in all phases of the framework and the continuous dialogue with Indigenous communities are logical.

Based on the informants’ input, some examples of the future use of this new framework follow: Creating supportive environments can lead to improvements in Indigenous women's clinical and social outcomes, which in turn can result in the development of personal skills. By redesigning the organization of health services in conjunction with implementing interventions to strengthen community actions, it would potentially overcome administrative barriers in healthcare management, increase the operationalization of care delivery, and enhance the efficiency, effectiveness, and quality of the healthcare network. Although the action “build healthy public policies” is part of the Canadian PHPM, it is absent from the original conceptual framework presented in this article.

Finally, one should be reminded that a model/framework is a graphic representation of the reality proposed by the researcher, because it has no limitations and is created within a constructivist perspective. The designed framework represents our perception (6 of us are Brazilians) of this social reality as lived by Brazilian Indigenous communities. Since the Canadian PHPM proposes in general terms integrated actions for health promotion, the reader can expect that PHC strategies to be conceived may be object of further refinement in future research implemented by graduate students affiliated to the main project.

## Discussion

Health promotion actions for the Indigenous Peoples should be based on humanistic, cultural, political, and moral principles within an emancipatory perspective, and be sensitive and responsive to traditional health sources (Zanchetta et al., 2016) and to the connection with “Mother Earth” (Mundel & Chapman, 2010). This connection is expressed, for example, by Canadian Indigenous Peoples in the idea of balance and harmony within and among dimensions of social, spiritual, emotional, and physical health ((Zanchetta et al., 2016).

Historically and contemporarily, socially in the Indigenous context, the experiences of Indigenous women have been neglected by the instrumental and positivist logic that relegates them to positions of social inferiority and subordination to traditional male hegemony (Pantoja, 2021). An unfavorable context and disrespect for these women's human rights results in precarious socioeconomic conditions and limited access to health services for them, which results in greater vulnerability, lower coverage of prenatal services, and difficulties accessing hospital childbirth in case of need (Figueiredo et al., 2024; Fundação Nacional de Saúde [FNS], 2002).

Reduced access to health services is due to a range of factors including language barriers, (causing feelings of isolation and anxiety particularly due to the inability to express personal concerns or preferences), limited availability of culturally sensitive care, especially in the context of maternal care, misinterpretation of treatment options, and limited understanding of informed consent and the clinical importance of the recommended follow-up (Rugumisa, 2024).

It is important to remind that the consultation did not identify content regarding the proposal for the development of specific public policies for the Indigenous women's population. This may be explained by the fact that Brazil has extensive assistance policies for maternal and child health aimed at the Indigenous population, implemented through continuous actions at various levels of care by the Unified Health System itself. We mention the Humanization of Labor and Birth Program (MS, 2000) and the National Policy for Healthcare of Indigenous Peoples (FNS), without the latter proposing specific actions and policies for the promotion of health for Indigenous women during the pregnancy and postpartum cycle.

Since the 2000s, there has been policy progress towards meeting the health needs of Indigenous women as well, such as the National Policy for Health Promotion (MS, 2010), the National Policy for Comprehensive Women's Health Care (MS, 2011), and the National Policy for Comprehensive Child Health Care (MS, 2015). The existence of these policies may explain the informants lack of comments regarding the need for other specific policies for Indigenous maternal health.

This article contributes to the advancement of conceptual knowledge regarding the promotion of Indigenous maternal health by presenting the perspectives of health professionals concerning the broader context of factors related to the pregnancy-puerperium cycle of Indigenous women. Such conceptual knowledge guides action research under implementation as an appropriate approach to promote social change and improve the quality of life of Indigenous women involved in the process. This can occur through the involvement and collaboration between researchers and participants working to identify important issues and develop strategies to address them (Bergamaschi & Pinheiro, 2021; Busarello & Sawaia, 2022).

New perspectives for rethinking ideas for modifying the identified situations may include social technologies and social responsibility in health. Social technologies need to be understood and integrated into a complex system of interactions between society and technology, reshaping social norms. Within the context of our action, social technologies are fundamental tools for rethinking ideas and fostering evolutionary processes capable of influencing the predominant social values of modifiable situations under social vulnerability. Under social technologies, inspiration, researchers and community members can study, analyze, and present methods to seek responsible innovation (Li et al., 2023).

Strategies focused on social innovation may help to address challenges within Indigenous populations by fostering a renewed understanding of underlying causes and by supporting the development of new models of Indigenous healthcare that improve access and enhance quality. It can be inspired by the concept of social responsibility in health (Réseau international francophone pour la responsabilité sociale en santé [RIFRS], 2025) that highlights the awareness of SDH and the priority needs of populations, which are essential aspects for the development of effective policies in specific contexts, such as among Indigenous populations. It can be operationalized in what RIFRS (2025) calls as the “Partnership Pentagon”. The idea of a pentagon represents the integration among policymakers, public authorities, health managers, academic institutions, and civil society (RIFRS, 2025). It can be inspirational to enable a multidimensional approach to improving maternal healthcare and reducing inequities among Indigenous women.

Regarding the gestational and postpartum cycle of Indigenous women, we can ponder that if such conceptual framework may be informative for creating effective strategies that appropriately meet their priorities. Moreover, such type of partnership model would inform new models of care whose implementation of public policies must be closely monitored by all involved to ensure their effectiveness. In this way, the interaction between social responsibility in Indigenous women's health and the identified contextual issues indicates the following: the needed collaboration among various social actors and consideration of local, cultural, and economic specificities are fundamental for improving health conditions and reducing inequalities in care for Indigenous mothers.

## Conclusion

The consultation revealed various forms of capital within Indigenous communities and the conditions, contradictions, and challenges caused by social inequalities. It also highlighted the sociocultural specificities of Brazilian Indigenous women's experiences during the pregnancy-puerperal cycle. The conception of AR could lead to the implementation of more effective and sustainable public policies, promoting real improvements in the quality of life of Indigenous women and their communities. This conceptual perspective on health promotion would harmonize technological and social innovations, mobilizing talents, skills, and scientific, popular, and traditional knowledge in Indigenous health.

While analyzing the gathered information within the perspective of the Canadian PHPM, we acknowledged that the Indigenous health system in Brazil has been significantly reorganized over the past two decades, but health indicators for pregnant women / new mothers continue to be unfavorable. A range of systemic situations compromising the quality of healthcare for Indigenous women, as described in the introduction and literature review in this article, served also as the basis for further conceptual reflection and development. Thoughts about the conceptualization of why, where, how, and with whom to seek possible socially responsive and innovative answers. Answers that are politically acceptable, economically viable, technologically and socially feasible, and culturally respectful.

Research projects are currently in implementation by Brazilian graduate students who are using the lens of the proposed conceptual framework while entering the field to then implement the data collection strategies herein suggested in coordination with Indigenous social-cultural leaders. The goal is an approximation with the everyday reproductive and maternal health experiences of Indigenous women. This fieldwork began in March 2025 and will extend until December 2027. The ongoing AR, which integrates multiple projects, seeks to collectively develop pathways to improve the health of Indigenous mothers, by promoting community leadership, respect for traditional knowledge, and contextualized and sustainable interventions.

This work contributes to the conceptual and methodological advancement in the promotion of Indigenous maternal health by proposing an original conceptual framework based on the integrative action areas of the Canadian PHPM and is congruent with Brazil's SUS and its consolidated National Policy for Indigenous Peoples Health. The analysis of the modifiable situations identified from the consultation highlighted the sociocultural specificities experienced by Indigenous women during the pregnancy-puerperal cycle under the influence of SDH.

The research fieldwork in progress can draw on social technologies and social responsibility in health as key instruments to foster social innovation and transform institutional practices, ultimately generating culturally sensitive outcomes and products. The development of such products is the key outcome expected by cohorts of Brazilian graduate students starting in January 2025 and ending in December 2027, who are coming to the TorontoMetropolitan University*, *University of Saskatchewan*, *and the Université d’Ontario Français for a research stay in knowledge translation (KT) and knowledge transfer (KTs), under the supervision of a multidisciplinary team of researchers.

The AR evolves toward the development of KT and KTs products takes into account the points of view of multiple social actors toward incorporating a multidimensional partnership model in Indigenous communities. This process involves the participation of public managers, health professionals, academic institutions, Indigenous leaders, and civil society. The ultimate outcome here is to propose new culturally appropriate care arrangements based on equity and social justice. This proposed conceptual framework offers a useful operational foundation for designing future interventions in Indigenous maternal health and for strengthening action research as a transformative methodological strategy in contexts marked by persistent historical and social inequalities.

Despite being grounded on Brazil's Indigenous maternal health context, this consultation can be inspirational to similar exploratory work by other researchers and practitioners interested in this topic, working collaboratively with natural experts in Indigenous health, whether they are health professionals or Indigenous individuals holding enough experiential knowledge to be considered as cultural insiders or culturally sensitive professionals.

## Data Availability

Not applicable.
